# Traumatic Isolated Lumbosacral Spinal Subdural Hematoma in a Pediatric Patient: A Case Report and Literature Review

**DOI:** 10.7759/cureus.93980

**Published:** 2025-10-06

**Authors:** Alexander Torres-Rey, Esteban R Rivera, Luis E Garcia-Irizarry, Samuel Estronza, Emil A Pastrana

**Affiliations:** 1 Emergency Medicine, Centro Médico Episcopal San Lucas, Ponce, PRI; 2 Neurosurgery, University of Puerto Rico, Medical Sciences Campus, San Juan, PRI; 3 Radiology, Comprehensive Cancer Center of Puerto Rico, San Juan, PRI

**Keywords:** inverted mercedes benz sign, pediatric, spinal hematoma, spinal subdural hematoma, trauma

## Abstract

Spinal subdural hematomas (sSDHs) are rare, particularly in the pediatric population and in the context of trauma without coexisting intracranial pathology. The pathophysiology of isolated sSDH is poorly understood, given the paucity of vasculature in the spinal subdural space. We present the case of a 16-year-old female who developed progressive lower back pain and difficulty with ambulation one week following traumatic ejection from a golf cart. Initial evaluation, including whole-body CT, revealed no abnormalities. One week after the initial trauma, the patient presented with back pain, limiting ambulation without focal deficits or signs of cauda equina syndrome. Magnetic resonance imaging demonstrated a subdural fluid collection from L1-S2 spinal levels and “inverted Mercedes-Benz” sign consistent with sSDH. The patient was managed conservatively with steroids and close observation. At her three-month follow-up, the patient had complete symptom resolution, and MRI/MRA demonstrated interval resolution of sSDH and no evidence of vascular malformations. A review of the literature identified six previous cases of traumatic isolated sSDH in pediatric patients. The majority presented with neurological deficits and were managed surgically. Our case represents the first reported instance of an isolated lumbosacral sSDH in a female pediatric patient managed successfully with conservative management. Isolated sSDH is exceedingly rare, particularly in the context of pediatric trauma, and may have a delayed presentation with no focal neurological deficits. MRI imaging is essential for diagnosis, and characteristic signs, such as the “inverted Mercedes-Benz” sign, may aid diagnosis. Conservative management may be an effective strategy in select cases of sSDH, such as those with stable, improving, or non-focal symptoms and no signs of hematoma expansion on imaging, highlighting the importance of individualized management strategies.

## Introduction

Spinal hematomas, including subarachnoid, subdural, and epidural hematomas, are rare but potentially life-threatening causes of spinal cord compression and neurological decline. Among these, epidural hematomas represent the most common subtype, followed by subarachnoid and subdural hematomas [1]. Spinal hematomas have been found to be associated with preexisting conditions, including hematologic disorders and vascular malformations, as well as the use of anticoagulation, trauma, and spinal procedures such as lumbar punctures and spine surgery [1].

Spinal subdural hematomas (sSDHs) occur infrequently and only a small proportion of sSDHs occur following trauma, making traumatic sSDHs particularly rare [1, 2]. The rarity of sSDHs may be attributed to the paucity of vasculature in the spinal subdural space [2]. The mechanism of subdural hematoma formation at this level is therefore unclear in the absence of vascular malformations. One theory is the migration of cranial subdural hematoma (cSDH) contents to the spinal subdural space due to the effects of pressure or gravity [3]. An alternative hypothesis posits that bleeding from ruptured radicular vessels into the subarachnoid space may subsequently track into the subdural compartment [4]. However, these mechanisms have yet to be definitively proven.

The clinical presentation of sSDH typically arises from acute compression of the spinal cord, manifesting as back pain, motor or sensory deficits, and, in some cases, cauda equina syndrome. Neurological symptoms often correspond to the level of the hematoma [1]. Despite sSDH representing a rare cause of spinal cord compression, prompt diagnosis and adequate treatment are imperative in order to avoid irreversible spinal cord injury and neurological deficits [1-2]. The most common treatment for sSDH is surgical intervention with laminectomy and durotomy for hematoma evacuation [2]. Despite limited literature, previously reported cases suggest conservative management may be a viable alternative in patients with no focal neurological deficit, stable neurological exams, and no radiographic signs of hematoma expansion [2,5-7].

In the United States, over five million children per year experience unintentional trauma that requires evaluation in the ED, and trauma remains the leading cause of death in children one to 18 years of age [8]. With trauma being a possible etiology of sSDH, further investigation and reporting of sSDH in the pediatric trauma setting is merited. In this report, we present a case of a 16-year-old female patient diagnosed with an isolated lumbosacral sSDH following a traumatic injury, highlighting the diagnostic challenges and successful conservative management. In addition, we present a review of the literature highlighting cases of traumatic sSDH in pediatric patients without concomitant intracranial pathology.

## Case presentation

A 16-year-old female presented to the emergency room with complaints of back pain and limited ambulation. The patient had no history of pre-existing medical conditions and took no medications but had experienced a recent traumatic accident. As per the patient's report, the patient was the passenger in a golf cart when the driver lost control of the cart. The patient was subsequently ejected from the vehicle, losing consciousness for approximately one minute. Following the accident, she was taken to an outside hospital emergency department (ED), where physical examination, full-body CT, and X-ray imaging noted no abnormalities.

**Figure 1:**
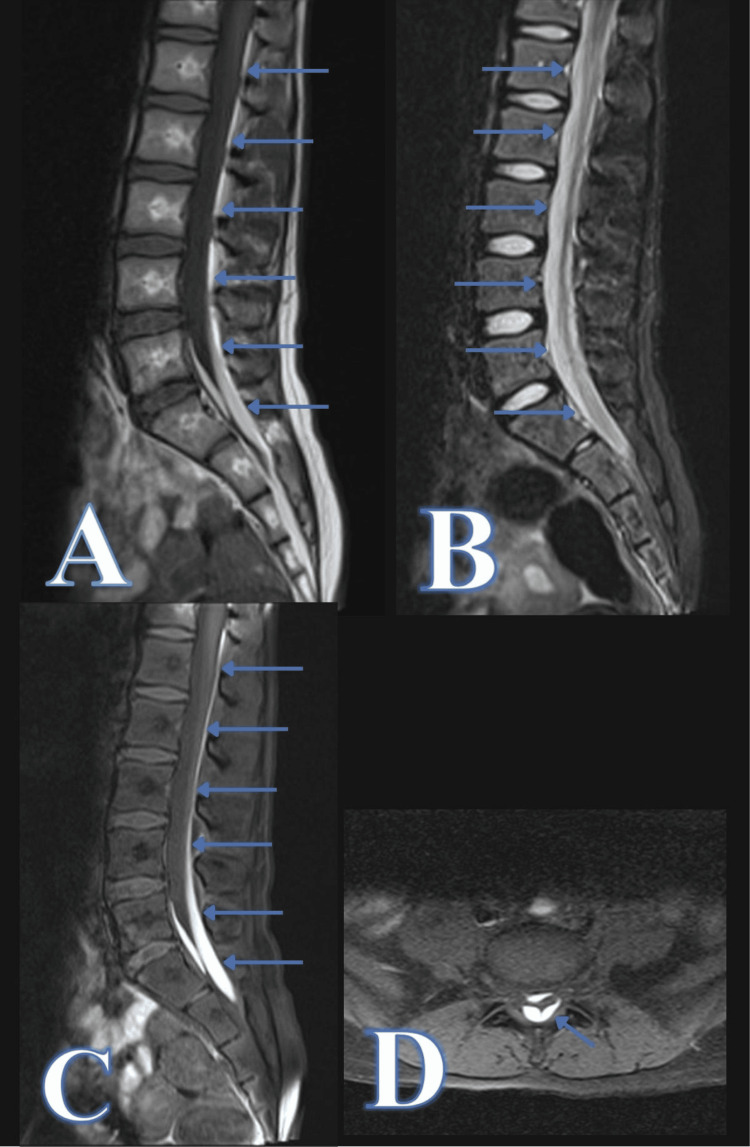
Diagnostic lumbo-sacral magnetic resonance imaging demonstrating a spinal subdural hematoma at L1-S2 levels. Sagittal T1 without fat suppression (A), sagittal short tau inversion recovery (STIR) (B), sagittal T1 with fat suppression (C), and axial T1 with fat suppression (D).

A week after the initial insult, the patient began experiencing significant back pain, which limited ambulation, and she presented to our ED. Given the history of trauma, an MRI was performed, which revealed a fluid collection along the anterior and posterior subdural spaces from L1-S2. The fluid collection demonstrated increased signal in both sagittal short tau inversion recovery (STIR) and T1 with fat suppression, favoring blood products (Figure 1). The patient was then transferred to our institution for pediatric neurosurgery evaluation and further management. 

On our evaluation, the patient was alert, awake, and oriented to person, time, and space. She reported bilateral lower extremity pain, which caused difficulty with ambulation. She denied leg paresthesia, urinary incontinence, saddle anesthesia, or difficulty with voiding or stooling. On physical examination, no deformities, lacerations, vertebral step-off, or tenderness of the spine were noted. There was no evidence of atrophy, fasciculations, or involuntary movements. Motor strength was 5/5 in all extremities. Sensation to light touch and pinprick, as well as reflexes, were globally intact. Laboratory testing confirmed no thrombocytopenia, electrolyte abnormalities, or coagulopathies. CT and CTA of the head, cervical, thoracic, and lumbar spines were performed and showed no evidence of vascular abnormalities or tumors. 

In the absence of neurological deficits suggestive of spinal cord compromise, a non-operative approach with conservative management was elected. A single dose of 4 mg dexamethasone was administered intravenously in the ED, and the patient was placed on bed rest with close observation for 24 hours. Following the 24-hour observation period, the patient was re-evaluated. No neurological deterioration was noted, and significant subjective improvement of pain was reported. The patient was subsequently discharged home on oral prednisolone.

**Figure 2:**
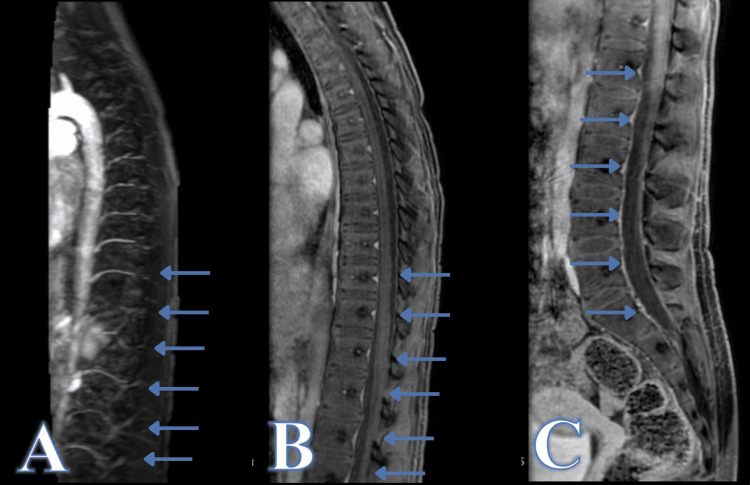
Magnetic resonance imaging demonstrating hematoma resolution and absence of vascular malformations Sagittal image of spinal magnetic resonance angiography (MRA) with IV contrast (A), sagittal T1 with fat suppression and IV contrast at the level of the thoracic spine (B) and sagittal T1 with fat suppression and IV contrast at the level of the lumbar spine (C).

The patient underwent a full spine magnetic resonance angiography (MRA) in the outpatient setting three months after the initial trauma. On evaluation, the patient was neurologically intact. MRI/MRA demonstrated interval resolution of previously noted blood products, with no evidence of a discrete nidus or shunting to suggest an underlying vascular malformation or tumor (Figure 2).

## Discussion

Isolated sSDHs are a rare complication of spinal trauma. Nonetheless, clinical suspicion of sSDH should be raised if symptoms arise days after the initial trauma [4]. Diagnosis of this rare pathology relies on imaging, with MRI being the modality of choice for spinal hematomas [9]. Several authors have described the “inverted Mercedes-Benz sign” as a characteristic imaging feature of sSDH [9-12]. The presence of this sign in our patient’s imaging further supports its utility as a diagnostic marker for sSDH.

There is no definitive consensus on the mechanism of sSDH formation. This is particularly noteworthy given the relative lack of blood vessels in the spinal subdural space, unlike the intracranial compartment. In addition, in the context of trauma, it is challenging to confirm isolated spinal injury due to the high likelihood of concomitant head trauma [13].One hypothesized mechanism is the migration of cranial subdural blood, primarily influenced by gravity [14]. This is likely the mechanism of sSDH whenever cSDH and sSDH are present simultaneously. This is supported by many cases of sSDHs that occur concomitantly with intracranial pathologies in non-accidental trauma. A series by Koumelis et al. involving 18 children reported that 44% of infants with non-accidental head trauma had spinal subdural collections, along with subdural hematomas in the supratentorial and infratentorial compartments [15]. In addition, there have been numerous reports of concomitant cSDH and sSDH [14-23], as well as cases in which sSDH appeared shortly after cSDH [24-26]. Alternatively, sSDH may arise from spinal hemorrhage, either spontaneously or secondary to trauma, vascular malformations, or anticoagulation [1]. We can therefore classify sSDHs by their likely mechanism as either migratory or isolated.

A review of the literature related to traumatic isolated sSDHs in the pediatric population was performed. The PubMed/MEDLINE case report database was searched utilizing the search parameters “pediatric spinal subdural hematoma”, “pediatric traumatic spinal subdural hematoma”, “pediatric traumatic cervical subdural hematoma”, “pediatric traumatic thoracic subdural hematoma”, “pediatric traumatic lumbar subdural hematoma”, and “pediatric lumbar subdural hematoma”. Additional papers were queried utilizing the search parameter "hematoma, subdural, spinal" (MeSH) and by cross-referencing cited articles. Articles addressing spontaneous and iatrogenic cases, as well as those involving hematologic disorders, vascular malformations, tumors, lumbar punctures, spinal epidural anesthesia, spinal or cranial surgery, or recent/concurrent cSDH, were excluded.

A total of six cases met the inclusion criteria, consisting of five males and one female (Table 1). The patients’ ages ranged from three to 20 years old, with a mean age of 10.7 years. Anatomical distribution included three cervical, two thoracic, and one lumbar SDH case. The most common presenting symptom was lower extremity weakness or paralysis, reported in five of the six cases. Four cases were managed with a laminectomy and durotomy for hematoma evacuation, and two were managed conservatively. Three cases experienced resolution of symptoms, one had partial recovery, and one showed limited improvement [6,7,27-30].

**Table 1:** Cases of traumatic isolated spinal subdural hematoma in pediatric population reported in the literature.

Age and gender	Injury level	Neurological deficits at presentation	Management	Outcome	Author, year of publication
8 y/o male	Cervical C1-C2	Local Neck Pain	Conservative	Symptom resolution	Aydin 2006 [6]
9 y/o male	Cervical C1-C2	Quadriplegia	Cervical laminectomy	Quadriplegia	Paredes, 1981 [27]
7 y/o female	Thoracic T2-T4	Back pain and paraplegia	Thoracic laminectomy	Resolved two weeks post-surgery	Z. Kotwika, 1989 [28]
3 y/o male	Thoracic T1-T11	Lower extremity paraplegia	Thoracic laminectomy 6, 7, 8	Lower limb diplegia	U Ozcan, 2002 [29]
20 y/o male	Lumbar L3-L5	Bilateral lower extremity paresthesia and toe paralysis	Conservative	Resolved 14 days post-trauma	Sudo H, 2012 [7]
17 y/o male	Cervical C4-C5	Left Hemiplegia	Cervical laminectomy	Left arm weakness	Kim, 2015 [30]
16 y/o female	Lumbosacral L1-S2	Lower back pain limiting ambulation	Conservative	Resolved 10 days post-trauma	Our case

The case presented in this report is of particular interest, as it represents the first reported pediatric case involving a female patient with isolated lumbosacral sSDH in the literature. The choice of whether to surgically intervene in our patient or manage conservatively was based primarily on the clinical finding of mild symptomatology without worsening of symptoms, similar to previously reported cases managed conservatively [6,7]. An additional consideration worth noting is the possible implementation of serial imaging to monitor subdural hematoma expansion, in which case surgical evacuation may be merited. In the present case, corticosteroids were employed, drawing from spinal compression literature suggesting that the use of corticosteroids may decrease inflammation and secondary injury to the spinal cord, therefore preventing further neurological impairment and alleviating pain [31]. The complete resolution of the patient’s symptoms, along with the interval resolution of the sSDH, suggests that conservative management may be a viable option for patients with isolated sSDH and no focal or progressing neurological deficits.

## Conclusions

Isolated sSDHs are exceedingly rare, particularly in pediatric patients and in the absence of concomitant intracranial pathology. This case contributes to the limited body of literature on sSDH by presenting the first reported instance of traumatic isolated lumbosacral sSDH in a female pediatric patient. In cases of traumatic isolated sSDH, symptoms may have a delayed onset, emerging days after the initial trauma. MRI with characteristic findings such as the “inverted Mercedes-Benz” sign may aid in early diagnosis. The favorable outcome in this case suggests that conservative management may be a viable alternative to surgical intervention for patients with isolated sSDH with mild symptoms, no focal neurological deficits, stable neurological exam, and no radiographic signs of hematoma expansion.
